# Endometriosis Beyond the Pelvis: A Case of Pancreatic Involvement

**DOI:** 10.7759/cureus.70530

**Published:** 2024-09-30

**Authors:** Adan Naseer, Boyd M Davis, Sara A Serji

**Affiliations:** 1 Internal Medicine, Eastern Virginia Medical School, Norfolk, USA

**Keywords:** endometriosis, endometriosis pancreatitis, extra-pelvic endometriosis, extrauterine endometriosis, pancreatitis

## Abstract

The prevalence of dysmenorrhea, the pain while menstruating, is high. Although most cases are benign, there should be a high suspicion of other etiologies such as endometriosis with recurrent episodes. Inaccurate or delayed diagnosis and treatment of endometriosis can lead to increased hospital visits and costs. We report the case of a 46-year-old female with a history of frequent hospital visits due to dysmenorrhea and menorrhagia, presented with clinical, laboratory, and image findings of pancreatitis with biopsy revealing endometriosis. This case report aims to highlight pancreatic endometriosis, a different and rare cause of pancreatitis, which should be suspected in cases of recurrent hospital visits with recurrent menstrual pain and abnormal presentation of pancreatitis.

## Introduction

Pancreatic endometriosis is a rare type of endometriosis in which ectopic endometrial tissue is found in the pancreas and could cause pancreatitis. Managing endometriosis in the emergency room setting is challenging due to how common it is, affecting 10%-15% of all women of reproductive age and 70% of women with chronic pelvic pain [1]. This complexity is heightened when the presence of ectopic endometriosis in uncommon locations like the pancreas, as it often mimics other more common pancreatic diseases such as chronic pancreatitis or pancreatic neoplasms. The clinical presentation is nonspecific and further complicates timely identification, typically presenting with abdominal pain. Imaging modalities, such as MRI or CT, may reveal pancreatic pathology, but pancreatic endometriosis relies on histopathological examination [2,3]. Understanding the pathophysiology of endometrial tissue migration and implantation within the pancreas is crucial for effective management. Only a handful of reported cases exist, and it can be missed on the differential. This case report highlights symptoms, diagnosis, and management of pancreatic endometriosis.

## Case presentation

**Figure 1:**
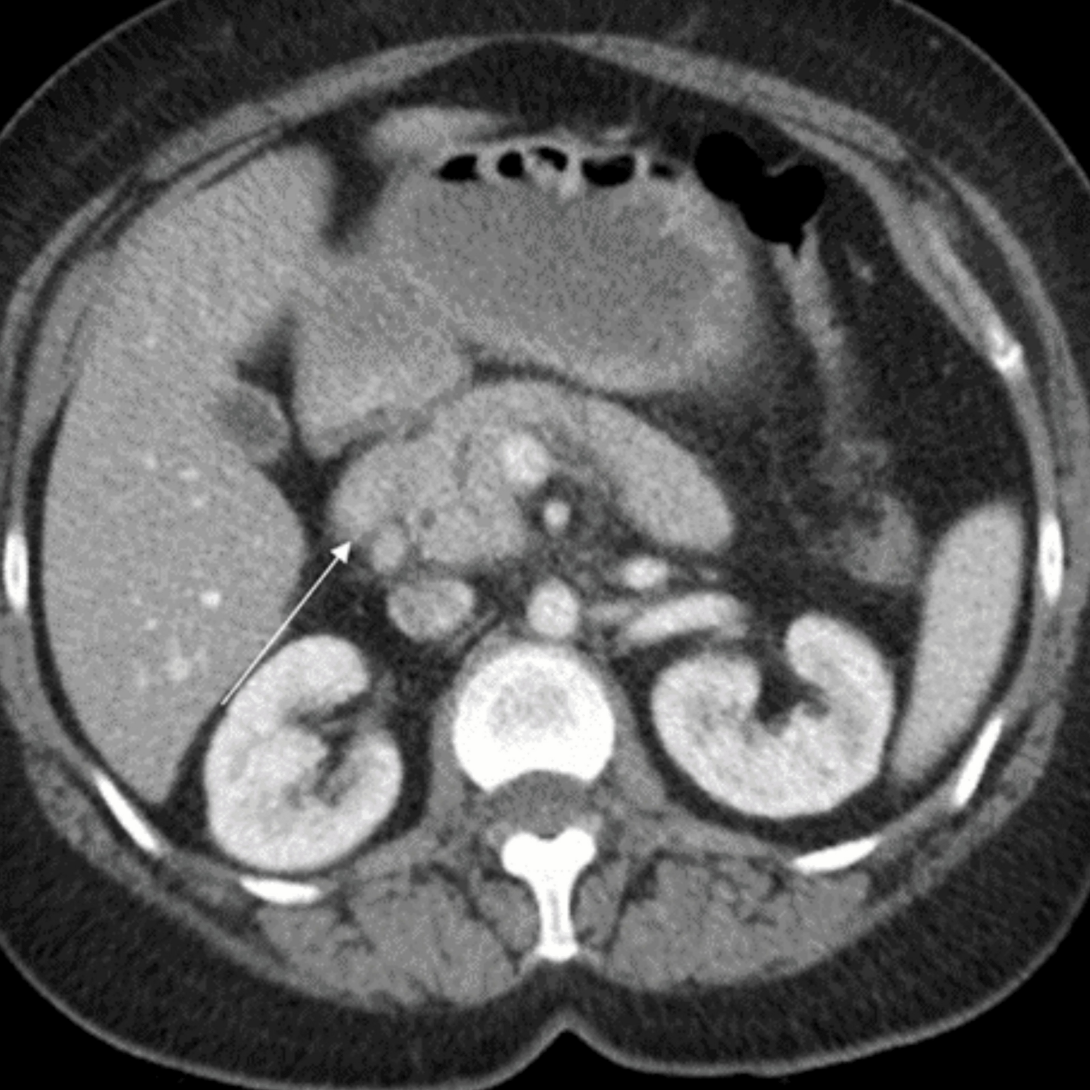
CT abdomen/pelvis showing mild acute interstitial edematous pancreatitis at the pancreatic head and uncinate process.

The patient is a 46-year-old female with a past medical history of heart failure with mildly reduced ejection fraction, hypertension, and type II diabetes who presented to the emergency department with the chief complaint of mid-epigastric pain. The patient started her menstrual cycle and began having pain two days prior to presentation. She reported similar pain associated with her menstrual cycle in the past. Her age of menarche was 16, and an obstetric history of gravida 2, para 2. Heavy periods started in her 30s, and she soaks through five to seven pads per 24 hours. She had at least nine repeated admissions every month during menstruation for the past year and also complained of nausea. She denies issues with urination or bowel movements. She also denied chest pain, shortness of breath, fever, decreased oral intake, or dizziness. She denied trauma or any use of steroids, tobacco, alcohol, or illicit drug use. Her only medicine is hydrochlorothiazide. On arrival, she was afebrile but hypertensive with a blood pressure of 209/104 and mildly tachycardic with a heart rate of 112/minute. She was tearful during the exam due to abdominal pain. Her abdomen was diffusely tender on palpation, primarily in the epigastric region, and bowel sounds were present. Complete blood cell count, and blood metabolic panel were unremarkable, except for an elevated lipase at 1,686. Her most recent triglyceride was elevated at 252. Ultrasound of the abdomen showed no gallstones. CT abd/pelvis with IV contrast revealed mild acute interstitial edematous pancreatitis at the pancreatic head and uncinate process (Figure 1). Pelvic and transvaginal ultrasound showed multiple intramural myometrial masses consistent with uterine fibroids but no ultrasound evidence of endometriosis. Since the patient had multiple ED visits every month with the same abdominal pain complaint associated with her menstruation cycle, endoscopic retrograde cholangiopancreatography (ERCP) was performed with a fine needle biopsy of the pancreatic tail which was consistent with endometriosis.

In the ED, she received IV fluids, multiple hydromorphone injections, ketorolac, and morphine. Her pain persisted, and she was ultimately admitted for pain control. OB/GYN was following inpatient and recommended outpatient hysterectomy and bilateral oophorectomy plan. The patient also received a leuprolide injection to induce premature menopause for endometriosis treatment. Throughout her admission, the patient slowly progressed with her diet, and pain slowly improved while on a PCA pump. She was transitioned to oral hydromorphone 2-4 mg q4-6 hours as needed for pain. She tolerated this transition well and was able to progress her diet to eat three meals without pain, nausea, or vomiting. While in the hospital, her outpatient hypertension medicine, hydrochlorothiazide, was switched to losartan 100 mg daily and Coreg 25 mg bid for concern of thiazide-induced pancreatitis. Her pain was much improved, and she was stable for discharge on day 5 with a pain regimen of 2 mg of oral hydromorphone q6h as needed for pain.

## Discussion

Endometriosis-induced pancreatitis is a rare diagnosis, with less than 20 cases reported in the literature. Endometriosis varies in presentation in women but often presents with dysmenorrhea, dyspareunia, dyschezia, dysuria, intermenstrual pain, and infertility [1]. It affects 10%-15% of all women of reproductive age [4]. One possible mechanism by which endometriosis occurs is through retrograde menstruation [5]. Several other theories have been proposed, such as lymphatic or vascular spread or celomic metaplasia [6]. The most common sites where endometriosis has been observed are in the pelvic organs, thus why this extra pelvic endometriosis presentation is rare. We propose that this case may indicate endometriosis is more likely caused by lymphatic or vascular spread or celomic metaplasia. The endometrial cells either had to travel a far distance in the abdominal cavity to reach the pancreas or arise in situ via metaplasia.

The variability of how endometriosis presents makes it not only difficult to diagnose but also to treat. Since abdominal pain is common with endometriosis, especially in a reproductive-aged woman, most would halt further evaluation. Therefore, we strive to increase awareness of this rare condition. The cyclical symptoms, history, and recurrent hospitalizations should require further investigation. Elevated lipase, along with abdominal pain and radiologic evidence of pancreatitis, prompts further evaluation with biopsy. However, endoscopic ultrasound-guided fine needle aspiration (EUS-FNA) should not be applied to cystic lesions based on fear of seeding that can cause pseudomyxoma peritonei [7]. While endometriosis is confirmed with a biopsy, depending on the affected area, other modes of imaging may be necessary [8].

Currently, there are no clear guidelines per the American College of Obstetricians and Gynecologists (ACOG) for managing extra pelvic endometriosis. A multidisciplinary approach is necessary. Management first includes suppression of ovarian function which can also aid in the diagnosis [9]. For example, leuprolide is a gonadotropin-releasing hormone analog that, at the administered dose, halts the secretion of gonadotropins. This leads to a decrease in steroidogenesis and less hormone-stimulated growth of endometrial cells. Otherwise, management of pain should be considered, with bowel rest and oral pain medicines. With extreme pain, fulguration should be considered [10]. Definitive treatment is only through surgery, necessitating thorough disease mapping to prevent residual disease [11]. But the most important thing is a preoperative diagnosis that would prevent an unnecessary resection in addition to significant morbidity and mortality that can happen [12].

## Conclusions

Pancreatic endometriosis is a rare etiology but should be considered as a differential in females with recurrent abdominal pain associated with their menstrual cycles. This case report highlights a rare case focusing on diagnosing and management. Increased awareness and clinical vigilance, along with a multidisciplinary approach, are advised to ensure timely diagnosis and enhance patient outcomes.
